# Vertically Oriented Quasi‐2D Perovskite Grown In‐Situ by Carbonyl Array‐Synergized Crystallization for Direct X‐Ray Detectors

**DOI:** 10.1002/advs.202309185

**Published:** 2024-05-13

**Authors:** Huiwen Chen, Ziyao Zhu, Bo Zhao, Weixiong Huang, Geping Qu, Zong‐Xiang Xu, Xue‐Feng Yu, Quanlan Xiao, Shihe Yang, Yunlong Li

**Affiliations:** ^1^ Materials Interfaces Center Shenzhen Institute of Advanced Technology Chinese Academy of Sciences Shenzhen 518055 China; ^2^ School of Materials Science and Engineering Anhui University of Science & Technology Huainan 232001 China; ^3^ Department of Chemistry Southern University of Science and Technology Shenzhen 518055 China; ^4^ International Collaborative Laboratory of 2D Materials for Optoelectronics Science and Technology of Ministry of Education Institute of Microscale Optoelectronics Shenzhen University Shenzhen 518060 China; ^5^ Guangdong Key Lab of Nano‐Micro Material Research School of Chemical Biology and Biotechnology Shenzhen Graduate School Peking University Shenzhen 518055 China

**Keywords:** orientation, perovskite quantum wells, Quasi‐2D perovskite thick films, well‐width distribution, X‐ray detectors

## Abstract

Quasi‐2D perovskite quantum wells are increasingly recognized as promising candidates for direct‐conversion X‐ray detection. However, the fabrication of oriented and uniformly thick quasi‐2D perovskite films, crucial for effective high‐energy X‐ray detection, is hindered by the inherent challenges of preferential crystallization at the gas‐liquid interface, resulting in poor film quality. In addressing this limitation, a carbonyl array‐synergized crystallization (CSC) strategy is employed for the fabrication of thick films of a quasi‐2D Ruddlesden‐Popper (RP) phase perovskite, specifically PEA_2_MA_4_Pb_5_I_16_. The CSC strategy involves incorporating two forms of carbonyls in the perovskite precursor, generating large and dense intermediates. This design reduces the nucleation rate at the gas‐liquid interface, enhances the binding energies of Pb^2+^ at (202) and (111) planes, and passivates ion vacancy defects. Consequently, the construction of high‐quality thick films of PEA_2_MA_4_Pb_5_I_16_ RP perovskite quantum wells is achieved and characterized by vertical orientation and a pure well‐width distribution. The corresponding PEA_2_MA_4_Pb_5_I_16_ RP perovskite X‐ray detectors exhibit multi‐dimensional advantages in performance compared to previous approaches and commercially available a‐Se detectors. This CSC strategy promotes 2D perovskites as a candidate for next‐generation large‐area flat‐panel X‐ray detection systems.

## Graphical abstract

The carbonyl array‐synergized crystallization strategy is used to reduce the nucleation rate of solution‐processed quasi–2D perovskites and passivate vacancy defects at the same time. The resultant quasi‐2D perovskite film, boasting a thickness exceeding half a hundred micrometers, demonstrates a vertically oriented structure and a pure well‐width distribution, thereby exhibiting commendable efficacy for direct X‐ray detection.

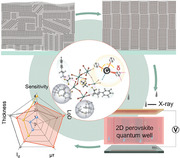

## 1 Introduction

Flat‐panel X‐ray detectors have facilitated the digital paradigm shift in X‐ray imaging within the context of the radiological revolution, assuming a pivotal role in the domains of medical imaging, security, and industrial flaw detection.^[^ ^1^, ^2^, ^3^ ^]^ Modern flat‐panel X‐ray detectors are in service with indirect conversion or direct conversion. The former uses a scintillator to convert X‐ray to visible light which in turn is converted to charges by a photodiode, while the latter uses an X‐ray photoconductor to convert X‐ray directly to electrons.^[^ ^4^, ^5^ ^]^ Benefiting from the absence of light scattering, the direct approach has a higher resolution. It is also believed with potential to be employed for advanced systems as well, for example, artificial intelligence.^[^ ^6^ ^]^ Nowadays, the most commercial X‐ray photoconductor of the direct X‐ray detector is amorphous selenium (a‐Se). However, it still has not been effectively promoted in the field of X‐ray imaging because of its inadequate absorption of hard X‐rays and high operating electrical fields.^[^ ^7^ ^]^ Recently, organic‐inorganic hybrid perovskites (OIHPs) have been demonstrated to be ideal X‐ray photoconductors due to their large mass attenuation coefficient, carrier mobility‐lifetime (µτ) product, and low‐cost solution fabrication process.^[^ ^8^ ^]^ Initially, 3D MAPbI_3_ photoconductors as well as photodiodes were manufactured to detect 8 keV soft X‐rays and obtained X‐ray sensitivity up to 1.1 µC Gy_air_^−1^ cm^−2^.^[^ ^9^ ^]^ After that, a series of OIHPs, for example, MAPbBr_3_, mixed‐halide perovskite single crystal, MAPbI_3_ thick film, wafer, etc. were fabricated for X‐ray detectors and showed the sensitivity record of 5.2 × 10^6^ µC Gy_air_^−1^ cm^−2^, which is extremely higher than commercial a‐Se.^[^ ^3^, ^7^, ^10^, ^11^, ^12^, ^13^ ^]^ However, 3D OIHPs suffer from serious ion migration problems and terrible moisture and chemical stability issues, and thus are limited in the application of commercial X‐ray detectors.^[^ ^14^ ^]^

2D and quasi‐2D OIHPs exhibit an inherent quantum well structure, characterized by organic spacers modulating octahedral sheets [PbI_6_]^2^^−^ These materials conform to a general formula of A’_2_A_n‐1_B_n_X_3n+1_ for the Ruddlesden‐Popper (RP) phase and A'A_n‐1_B_n_X_3n+1_ for the Dion‐Jacobson (DJ) phase. In this context, A’ denotes a long‐chain spacing ligand, A signifies a monovalent short‐chain ammonium cation, B represents a divalent metal ion such as Pb^2+^ or Sn^2+^, X represents the halide ion, and n indicates the number of the octahedral sheets [PbI_6_]^2^^−^ in the quantum well, corresponding to the well width.^[^ ^15^, ^16^ ^]^ Compared with 3D OIHPs, 2D and quasi‐2D OIHPs exhibit suppressed ion migration and higher structural stability due to the introduction of long‐chain spacers.^[^ ^8^ ^]^ These benefits landed 2D and quasi‐2D OIHPs increasing attention in the field of X‐ray direct detection.^[^ ^17^ ^]^ Many works on 2D and quasi‐2D OIHPs direct X‐ray detection with excellent performance focused on single crystals, in which Pb‐based perovskite delivered sensitivity from 2.42 × 10^2^ to 1.32 × 10^4^ µC Gy_air_^−1^ cm^−2^ and ultra‐low detection limit of 23 nGy_air_ s^−1^.^[^ ^8^, ^17^, ^18^, ^19^ ^]^ Meantime, it is found that the carrier transport is confined within the quantum well plane which causes anisotropy of X‐ray sensitivity. Although the artificial single crystals can promise exciting performance by fixing charge‐carriers transmission direction along with the orientation of the quantum well plane, they are still too small to compatible with flat panel X‐ray detectors. Solution‐based in‐situ orientated growth of crystalline film is an alternative method that can guarantee both requirements of orientation and large area. Tsai et al. demonstrated a thin‐film (≈500 nm) X‐ray detector comprised of highly crystalline and vertically oriented quasi‐2D OIHPs by the spin‐coated, possessing a volume sensitivity up to 0.276 C Gy_air_^−1^ cm^−3^.^[^ ^20^ ^]^ Due to the limit of thickness, the amount of charge generated per unit area (cm^−2^) of thin‐film x‐ray detector fabricated by Tsai et al. is only 0.138 µC Gy_air_^−1^ cm^−2^. The low sensitivity is ascribed to the ultralow absorption of the X‐ray by the thin perovskite film. It is found that the nucleation and growth of quasi‐2D OIHPs arise from the liquid–air interface,^[^ ^21^ ^]^ and then form a “shell” on the surface and inhibit precursor solvent volatilization, resulting in serious bulk and surface defects (e.g., pin‐holes and inhomogeneity). Thus, the fabrication of a quasi‐2D perovskite thick film with a high degree of orientation via solution‐based coating remains to be a big challenge. Currently, vertically oriented quasi‐2D perovskite films thicker than 10 µm have not been fabricated by solution in situ crystallization method.^[^ ^22^ ^]^

In this work, we propose a carbonyl array‐synergized crystallization (CSC) strategy by using both phenethylamine acetate (PEAAc) and polyvinylpyrrolidone (PVP) for in‐situ vertical growth of a quasi‐2D PEA_2_MA_4_Pb_5_I_16_ RP perovskite thick films via solution process. It is demonstrated that carbonyls in PEAAc and PVP synergistically induce the formation of large and dense intermediates via Lewis acid‐base interaction with Pb^2+^ and hydrogen bonding with ‐NH_3_^+^_,_ respectively. The intermediates constrained the methyl vibration and thus reduced the nucleation rate. In addition, the CSC strategy enhances the binding energies of Pb^2+^ on (111) and (202) planes. Therefore, the CSC‐based PEA_2_MA_4_Pb_5_I_16_ RP perovskite prefers vertical orientation, pure quantum well width as well as uniform morphology. Independently, carbonyls in PEAAc reduce the grain boundaries of the thick film whereas carbonyls in PVP passivate vacancy defects of cation and iodine, inhibiting the non‐radiative recombination of carriers. Correspondingly, both trap‐state densities of electron and hole decrease in CSC‐based PEA_2_MA_4_Pb_5_I_16_ RP perovskite thick films. These benefits lead to improved X‐ray sensitivity, limit of detection, and operational stability. Our work suggests an in situ crystallization method especially suitable for large‐scale printable X‐ray detectors via additives engineering which allows vertically oriented PEA_2_MA_4_Pb_5_I_16_ RP perovskites with pure well width. We believe that with further improvement in quality and thickness, the quasi‐2D perovskite thick film will be a strong candidate for next‐generation large‐area X‐ray imaging systems.

## 2 Results and Discussion

### 2.1 Structure and Morphology

For the carbonyl array‐synergized crystallization (CSC) strategy, phenethylamine acetate (PEAAc), lead iodide (PbI_2_), and methylammonium iodide (MAI) were mixed at an appropriate stoichiometric ratio in the binary solvent of dimethylformamide (DMF) and dimethyl sulfoxide (DMSO), subsequently, polyvinylpyrrolidone (PVP) was added to prepare quasi‐2D PEA_2_MA_4_Pb_5_I_16_ (n = 5) perovskite precursor, shown in Figure S1 (Supporting Information). To verify the synergistic contribution of PEAAc and PVP to in‐situ crystallization orientation of the PEA_2_MA_4_Pb_5_I_16_ RP perovskites, we prepared two controlled precursors, one removed PVP (labeled as c‐PEAAc) and the other removed Ac by replacing PEAAc with PEAI (labeled as c‐PVP). Afterward, the precursors were blade coated on the indium tin oxide (ITO) glasses, and then perovskite thick films were fabricated after the programmed drying and annealing process. Finally, the X‐ray detector was obtained by evaporating the Cu as the top electrode on the surface of the PEA_2_MA_4_Pb_5_I_16_ RP perovskite thick film. During the process of perovskite fabrication, we find that a long‐time treatment at low temperature is indispensable for highly‐crystalline film since a short‐time treatment is not enough to fully remove the solvent and invariably leads to low crystallinity (Figure S2, Supporting Information).

To determine the optimal dosage of PVP, X‐ray diffraction (XRD) patterns of CSC‐based perovskites with varying amounts of PVP were systematically investigated (Figure S3, Supporting Information). The XRD analysis of PEA_2_MA_4_Pb_5_I_16_ RP perovskites reveals three prominent peaks corresponding to the vertically oriented quasi‐2D organic‐inorganic hybrid perovskite planes of (111), (202), and (313). Evidently, the intensity of these dominant peaks exhibits a positive correlation with the added dosage of PVP. In CSC‐based perovskites with PVP ranging from 0.0 to 1.5 wt.%, diffraction peaks associated with low‐n 2D phases, including the (00*l*) planes of n = 1, 2, (0*k*0) planes of n = 3, 4, as well as randomly oriented phases such as the (0*k*0) planes of n = 5 and 3D phases, are consistently observed. Notably, when the PVP dosage reaches 2.0 wt.%, the diffraction peaks corresponding to these phases vanish, leaving only vertically oriented peaks of n = 5 discernible. Thus, the selection of a PVP dosage of 2.0 wt.% is deemed optimal to achieve PEA_2_MA_4_Pb_5_I_16_ RP perovskite with a vertical orientation and a pure well‐width distribution, laying the foundation for subsequent investigations.

**Figure 1:**
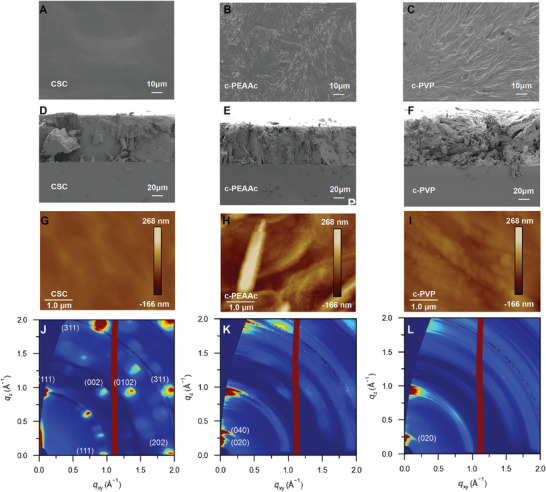
A–C) Top‐view SEM images CSC, c‐PEAAc, and c‐PVP‐based PEA_2_MA_4_Pb_5_I_16_ RP perovskite thick films. D–F) Cross‐section SEM images of CSC, c‐PEAAc, and c‐PVP‐based PEA_2_MA_4_Pb_5_I_16_ RP perovskite thick films. G–I) AFM images of CSC, c‐PEAAc, and c‐PVP‐based PEA_2_MA_4_Pb_5_I_16_ RP perovskite thick films. J–L) GIWAXS spectra of CSC, c‐PEAAc, and c‐PVP‐based PEA_2_MA_4_Pb_5_I_16_ RP perovskite thick films.

**Figure** **1**A,D show the scanning electron microscopy (SEM) images of surface and cross‐section of CSC‐based PEA_2_MA_4_Pb_5_I_16_ RP perovskite thick film, revealing a notably smooth surface and well‐defined cross‐section. Comparative analysis with c‐PEAAc (Figure 1B,E) and c‐PVP‐based samples (Figure 1C,F) reveals that PEAAc singularly contributes to the effective reduction of grain boundaries and enhancement of cross‐sectional morphology, while the addition of PVP alone exhibits no discernible impact. However, an examination of CSC‐based samples in conjunction with c‐PEAAc reveals that the supplementary addition of PVP contributes to an amelioration in surface morphology (Figure 1A,B; Figure S4, Supporting Information). It can be further confirmed by atomic force microscopy (AFM) (Figure 1G–I; Figure S5, Supporting Information), wherein the root‐mean‐squared (RMS) roughness of CSC‐based sample is reduced from 47.9 nm (c‐PEAAc) and 15.4 nm (c‐PVP) to 7.61 nm. Generally, PEAAc improves the cross‐section while PVP enhances surface morphology, both are indispensable for promoting morphology.

The photoluminescence (PL) spectra integrated from 500 to 850 nm for CSC, c‐PEAAc, and c‐PVP‐based PEA_2_MA_4_Pb_5_I_16_ RP perovskite thick films were characterized to further explore their microstructure (Figure S6A, Supporting Information). It is clear that c‐PEAAc‐based sample is dominated by low‐n emission peaks, that is, peaks at n = 1 (522 nm), n = 2 (567 nm), n = 3 (648 nm), n = 4 (674 nm), especially n = 1. While PVP tends to induce the formation of a high‐n phase, with emission peaks n = 4 (675 nm), n = 5 (758 nm), and n = ∞ (3D phase at 800 nm).^[^ ^23^, ^24^ ^]^ When PEAAc and PVP work synergistically, the PL emission peaks other than n = 5 disappear in the CSC‐based sample. Simultaneously, narrower full width at half maximum (FWHM) and higher intensity of the emission peak of n = 5 in the CSC‐based sample than those in c‐PEAAc and c‐PVP‐based samples further demonstrate that a purer‐phase and higher‐quality PEA_2_MA_4_Pb_5_I_16_ RP perovskite is formed by the synergistic induction of PEAAc and PVP. Compared to c‐PEAAc and c‐PVP‐based samples, the CSC‐based sample exhibits a pronounced blue‐shift of the peak of n = 5, attributed to the decrease of roughness, explained by previous work,^[^ ^25^ ^]^ further demonstrating that synergy of PEAAc and PVP is critical to improving surface morphology of the corresponding PEA_2_MA_4_Pb_5_I_16_ RP perovskite.

XRD patterns were acquired to reveal the crystallization quality of PEA_2_MA_4_Pb_5_I_16_ RP perovskite thick films as well, shown in Figure S6B,C (Supporting Information). The CSC‐based sample exhibits only vertically orientated quasi‐2D OIHPs planes of (111), (202), and (313), shown in Figure S6B (Supporting Information). In the c‐PEAAc‐based sample, various 2D phases of low n, (00*l*) plane of n = 1, 2, (0*k*0) planes of n = 2, 3, 4, and randomly orientated phases, (0*k*0) planes of n = 5, appear. Similarly, in the c‐PVP‐based sample, (0*k*0), and (00*l*) planes of various 2D phases with different n, accompanied by (112), (211), (202), (310), and (224) planes of 3D phase, are detected (Figure S6B,C, Supporting Information),^[^ ^24^, ^25^, ^26^ ^]^ which is consistent with the PL results. The simultaneous induction of PEAAc and PVP is key to realizing vertical orientation and high crystallinity, neither a single PEAAc nor PVP can achieve this result.

To conduct a comprehensive assessment of the orientation of CSC on the substrate, grazing incidence wide‐angle X‐ray scattering (GIWAXS) analysis was carried out on films based on CSC, c‐PEAAc, and c‐PVP (Figure 1J‐L). The diffraction patterns of the c‐PEAAc and c‐PVP‐based films displayed characteristic powder‐like rings, indicative of random orientation. Conversely, the CSC‐based film exhibited distinct and sporadic Bragg spots, indicating a highly vertically oriented growth pattern, which aligns with the findings from XRD patterns. Additionally, it's worth noting that the red line observed at ≈1.1 Å^−1^ corresponds to the detector gap. Simultaneously, the emergence of distinct (0k0) peaks at a narrow‐angle is observed, which is attributed to a blend of low‐n phases.^[^ ^27^ ^]^ This phenomenon is notably pronounced in films based on c‐PEAAc and c‐PVP. Remarkably, in the CSC‐based film, these peaks associated with low‐n phases are absent, indicating a synergistic effect between PEAAc and PVP that impedes the formation of low‐n phases and augments the phase purity of quasi‐2D perovskite. Furthermore, to characterize the top‐down orientation and crystallinity, we operated XRD of the CSC‐based thick films with different thicknesses (Figure S7, Supporting Information). The samples with thicknesses from 15 to 60 µm all exhibit three characteristic diffraction peaks of quasi‐2D vertically oriented perovskites (Figure S7A, Supporting Information), illustrating vertical orientation from top to bottom. However, with the increase of thickness, the FWHM of the diffraction increases and the intensity decreases, indicating that the crystallinity of thick films decreases.

### 2.2 Crystallization Mechanism

Fourier transform infrared spectra (FTIR) analysis was conducted to distinguish the function of each functional group in the precursor (Figure S8, Supporting Information). Initially, the PbI_2_ was dissolved in a mixed solvent of DMSO:DMF. It is found that the vibration of ─S═O of DMSO is significantly weakened and the vibration frequency of ─C═O of DMF is decreased, suggesting Pb^2+^ has a strong interaction with solvent through ─S═O and ─C═O. With the further addition of PVP, the bands at 1665, 997 and 946 cm^−1^ appear, corresponding to the stretching vibration of ─C═O, ─C─N, and ─C─O in PVP. The ─C═O of DMF shows a redshift after the addition of PVP. It is demonstrated that the electron of ─C═O is delocalized and the instantaneous dipole increase, revealing the carbonyl in PVP changes the electron distribution of ─C═O in the precursor. Meantime, compared to the FTIR spectrum of pure PVP, as shown in Figure S8B (Supporting Information), the FTIR spectrum of PVP with PbI_2_ shows that the stretching vibration of ─C═O at 1655 cm^−1^ in PVP undergoes a sudden decrease in intensity and a concomitant red‐shift, revealing that the ─C═O in PVP strongly interact with Pb^2+^, which then leads to a red shift of the stretching vibration of ‐C─N and ─C─O as well. Then bands of ─C═O started merging into a single one with the addition of the spacer salt, illustrating both PEAI and PEAAc can average the electron density of ─C═O, attributed to the hydrogen bond between ‐NH_3_^+^ and ─C═O. Finally, with the addition of MAI, the stretching vibration of ─C═O further merges, indicating that ‐NH_3_^+^ in MAI can also form the hydrogen bond with ─C═O. Notably, the hydrogen bond between ‐NH_3_^+^ and ─C═O can assist in the formation of large intermediates, which is beneficial to improve the crystallinity and vertical orientation of quasi‐2D perovskites.^[^ ^24^ ^]^ In addition, we find the bands of CSC precursor with PbI_2_ at 1658 cm^−1^ (─C═O), 1553 cm^−1^ (‐NH_3_^+^ in PEAAc), and 957 cm^−1^ (─C─O in PVP) are all red‐shifted compared to that of CSC precursor without PbI_2_, shown in Figure S8C (Supporting Information). It is believed that the redshift is derived from both the Lewis acid‐base interaction of ─C═O (in DMF, PEAAc, and PVP) with Pb^2+^ and the hydrogen bond of ─C═O with ‐NH_3_^+^ (in PEAAc) ^[^ ^28^, ^29^ ^]^ after combining the results of Figure S8A,C (Supporting Information).

**Figure 2:**
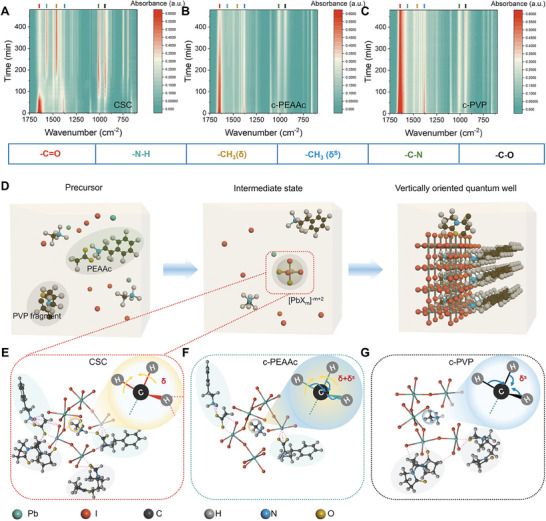
A–C) In situ IR spectra of the CSC, c‐PEAAc, and c‐PVP‐based samples during the first 8 h of crystallization. D) Schematic diagram of the crystallization process of CSC‐based sample. E–G) The intermediate structure, accompanied with methyl vibration of CSC, c‐PEAAc, and c‐PVP‐based samples.

To further investigate the crystallization process, in‐situ infrared spectra of the CSC, c‐PEAAc, and c‐PVP‐based samples within the first 8 h of crystallization were tested, respectively, as shown in **Figure** **2**A–C. In contrast with the c‐PEAAc and c‐PVP‐based sample, the stretching vibration of ─C═O of the CSC‐based sample is blue‐shifted, indicating the increasing electron density of ─C═O with the incorporation of PEAAc and PVP. With the passage of time, the vibration peak of ─C═O becomes weak obviously in the CSC‐based sample while has a minor change in c‐PEAAc and c‐PVP‐based samples, indicating that the Lewis acid‐base interaction of ─C═O with Pb^2+^ can be promoted with the synergy of carbonyl array in PEAAc and PVP. Simultaneously, the vibration of ‐N─H in the CSC‐based sample becomes strong gradually while no signal appears in c‐PEAAc and c‐PVP‐based samples, demonstrating the synergy of the carbonyl array can induce the formation of hydrogen bonds with ─N─H. Therefore, it is believed that larger intermediates are formed during the crystallization process in the CSC‐based sample, which inhibits the rapid nucleation of PEA_2_MA_4_Pb_5_I_16_ RP perovskite quantum wells (shown in Figure S9, Supporting Information), leading to higher crystallinity and purer well width distribution, as illustrated in Figure 2D,E.^[^ ^24^, ^30^, ^31^ ^]^ In addition, the symmetrical deformation vibration of ─CH_3_ (─CH_3_(δ^S^)) transforms to in‐plane asymmetric bending vibration (─CH_3_(δ)),^[^ ^32^, ^33^ ^]^ indicating dense intermediates are formed in the first 8 h of crystallization, which limits the symmetrical deformation vibration of methyl group and thus induces vertical orientation in CSC‐based samples. The c‐PEAAc‐based sample exhibits a similar transform of ─CH_3_ vibration to the CSC‐based sample, but to a lesser extent, indicating less dense intermediates formed and decline the orientation, shown in Figure 2F. As a comparison, the c‐PVP‐based sample barely shows a pronounced transformation to in‐plane asymmetric of methyl, due to the absence of the dense intermediate (Figure 2G), leading to the random orientation. In brief, the CSC strategy promotes ─C═O forming Lewis acid‐base interaction with Pb^2+^ as well as the hydrogen bond with ─NH_3_^+^, followed by the formation of large and dense intermediates, changing the vibration of methyl and reducing the nucleation rate, thus improving the surface topography, crystallinity, phase purity and orientation of PEA_2_MA_4_Pb_5_I_16_ RP perovskite.

To gain a deeper understanding of the orientation mechanism of CSC‐based PEA_2_MA_4_Pb_5_I_16_ RP perovskites, density functional theory (DFT) calculations were carried out. These calculations aimed to examine the binding energies of Pb^2+^ ions on two specific planes, namely (111) and (202), theoretically. This investigation is motivated by the association of the (111) plane with the tilted vertical orientation, while the (202) plane characterizes the optimal out‐of‐plane orientation.^[^ ^34^ ^]^ Initially, a theoretical model of PEA_2_MA_4_Pb_5_I_16_ was constructed according to the result of XRD, shown in Figure S10A,B (Supporting Information). The binding energies of Pb^2+^ on (111) and (202) planes were then calculated from the CSC and non‐CSC‐based PEA_2_MA_4_Pb_5_I_16_ RP perovskites (Figure S10C, Supporting Information), respectively. As shown in Figure S10D (Supporting Information), the CSC‐based PEA_2_MA_4_Pb_5_I_16_ RP perovskite exhibits significantly enhanced binding energies of Pb^2+^ on the two planes, compared to the non‐CSC‐based PEA_2_MA_4_Pb_5_I_16_ RP perovskite. This verified the preferred growth of vertical orientation along with [111] and [202] directions in the CSC‐based perovskite, which is consistent with previous XRD results (Figure S6). Furthermore, by combining the DFT calculations with the FTIR results, this study was able to conclude that CSC promotes the formation of large and dense intermediates through Lewis acid‐base interaction and hydrogen bonds, and enhances the binding of Pb^2+^ on (111) and (202) planes, thereby enhancing orientation.

### 2.3 Physical Properties of PEA_2_MA_4_Pb_5_I_16_ RP Perovskites

**Figure 3:**
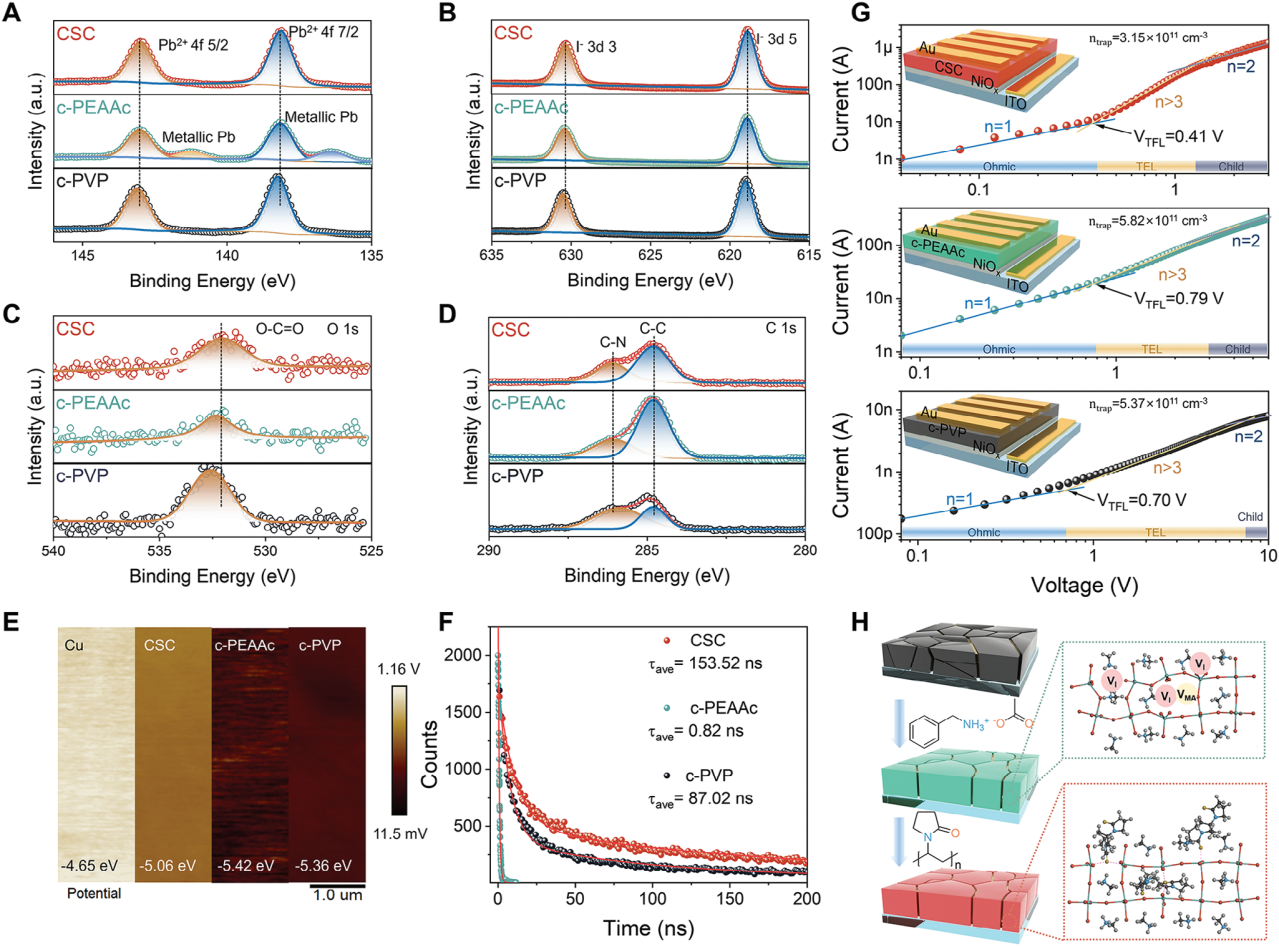
A–D) Pb 4f, I 3d, O 1s, and C 1s XPS spectra of CSC, c‐PEAAc, and c‐PVP‐based samples. E) KPFM data for CSC, c‐PEAAc, and c‐PVP‐based samples. F) TRPL spectra of CSC, c‐PEAAc, and c‐PVP‐based thick films. G) SCLC measurements of hole‐only devices based on CSC, c‐PEAAc, and c‐PVP samples. H) Schematic diagram of reducing vertical grain boundaries by PEAAc and passivating defects by PVP.

First, the UV‐Vis absorption spectra of CSC, c‐PEAAc, and c‐PVP‐based films were measured to assess the reaction of ions in the precursor, shown in Figure S11 (Supporting Information). No significant absorption peaks are observed at 510 nm, which is the characteristic peak of PbI_2_,^[^ ^35^ ^]^ revealing the complete reaction among the precursor ions. Then, the XPS was applied to investigate the chemical state of elements in the PEA_2_MA_4_Pb_5_I_16_ RP perovskite thick films prepared by CSC, c‐PEAAc, and c‐PVP‐based precursors (**Figure** **3**A–D). It is found that the binding energy peaks at 136.4 and 141.3 eV, which contributed to metallic Pb, exist in the c‐PEAAc‐based sample. However, the peaks disappear in the CSC and c‐PVP‐based samples. The metallic Pb is derived from cation and iodine vacancy defects, which can be suppressed by PVP since the carbonyl in PVP can form the Lewis acid‐base interaction with Pb^2+^.^[^ ^36^ ^]^ In addition, compared to those of c‐PEAAc and c‐PVP‐based samples, the Pb^2+^ 4f, I^−^ 3d, and O 1s peaks of CSC‐based sample shift toward lower binding energy. In contrast, the ─C─N peak of CSC‐based sample shifts toward higher binding energy. The opposite shift demonstrates the electrons in ─C─N transfer to I^−^, ─C═O, and Pb^2+^.^[^ ^37^, ^38^, ^39^ ^]^ The difference of ─C─N peaks between CSC‐based sample and the c‐PVP‐based sample is significantly greater than that between CSC‐based sample and c‐PEAAc‐based sample since the PEAAc promotes more electrons transformation from ─C─N to I^−^, ─C═O and Pb^2+^ than PVP, owing to the stronger interaction between─C═O and Pb^2+^ through Ac^−^.^[^ ^40^ ^]^ In brief, PVP can suppress metallic Pb and reduce vacancy defects while PEAAc can enhance the Lewis acid‐base interaction between Pb^2+^ and ─C═O, and then reduce the grain boundaries. The influence of carbonyls in PEAAc and PVP on the film formation of PEA_2_MA_4_Pb_5_I_16_ RP perovskite is further distinguished. It is worth mentioning that, according to the previous work, the ─C─N content in C 1s peaks of 3D perovskites is higher than that of PEA_2_MA_4_Pb_5_I_16_ RP perovskites.^[^ ^41^ ^]^ The ─C─N content in PEA_2_MA_4_Pb_5_I_16_ RP perovskite decreases with the increase of spacer concentration.^[^ ^42^ ^]^ The ─C─N content in c‐PVP‐based thick film is apparently higher than that in CSC‐based thick film since c‐PVP‐based thick film is mainly distributed with 3D perovskite phase, which is absent in CSC‐based thick film. Likewise, the ─C─N content in c‐PEAAc‐based thick film is lower than that in CSC‐based thick film, since the low n (n<5) phase is widely present in c‐PEAAc‐based thick film, while only the 2D phase with n = 5 exists in CSC‐based thick film. The phenomenon echoes the results of PL and XRD.

Then, Kelvin probe force microscopy (KPFM) was applied to identify the surface work function change affected by the synergy of the carbonyl array in PEAAc and PVP (Figure 3E). The surface work function was calculated from surface potential differences between Cu electrodes and PEA_2_MA_4_Pb_5_I_16_ RP perovskite thick films. Compared with the surface work function of c‐PEAAc‐ (−5.42 eV) and c‐PVP‐ (−5.36 eV) based thick film, the surface work function of CSC‐based thick film increases to −5.06 eV, which matches better with Cu electrode (−4.65 eV). A closer surface work function indicates better energy band alignment, which is beneficial to the carrier extraction at the interface of the electrode and PEA_2_MA_4_Pb_5_I_16_ RP perovskite thick film.^[^ ^43^ ^]^ The time‐resolved photoluminescence (TRPL) spectroscopy was performed to evaluate the carrier lifetime. As shown in Figure 3F and Table S1 (Supporting Information), the carrier average lifetime of CSC‐based sample (153.52 ns) is nearly 200 times higher than that of c‐PEAAc‐(0.82 ns) based sample and doubled higher than that of c‐PVP‐based sample (87.02 ns), indicating greatly suppressed non‐radiative recombination in CSC‐based sample. This is also proved by the steady PL spectra, where CSC‐based sample possesses much stronger emission intensity than c‐PEAAc and c‐PVP‐based samples at 750 nm (n = 5).

Finally, Hole‐only and electron‐only space‐charge limited current (SCLC) devices were fabricated to analyze the hole and electron trap state density (n_trap_), respectively. As shown in Figure 3G and Figure S12 (Supporting Information), ohmic and trap‐filled regions are clearly exhibited at low voltage. Ohmic region (n = 1) shows the ohmic response between the electrodes and the thick films, and trap‐filling region (n = 2) indicates that the injected carriers fill in the trap states. The kink point from ohmic to trap‐filling region defines the trap‐filled limit voltage (V_TFL_). The *n_trap_* can be determined by the Equation 1:

(1) $$n_{\mathrm{trap}}=\frac{2\varepsilon_{0}\varepsilon V_{\mathrm{TEL}}}{qL^{2}}\;$$

whereas ε_0_ refers to the dielectric constant, ε represents the dielectric constant in vacuum, q is the elemental charge, and L is the thickness.^[^ ^18^ ^]^ The hole n_trap_ values for the CSC, c‐PEAAc, and c‐PVP‐based samples are determined to be 3.15 × 10^11^, 5.82 × 10^11^, and 5.37 × 10^11^ cm^−3^, respectively. Concurrently, the calculated electron n_trap_ values for the CSC, c‐PEAAc, and c‐PVP‐based samples are 3.45 × 10^11^ , 6.11 × 10^11^, and 5.37 × 10^11^ cm^−3^, respectively. It is evident that both hole and electron n_trap_ values in CSC‐based samples are lower than those observed in the c‐PEAAc and c‐PVP‐based thick films. This discrepancy in trap densities indicates a favorable charge carrier transport and reduced recombination in the CSC‐based samples. In general, the PEAAc is employed to reduce vertical grain boundaries, since the strong interaction of carbonyl in PEAAc with Pb^2+^ reduces the nucleation rate and enhances the crystallinity in the vertical direction. However, the mere addition of PEAAc produces MA^+^ and I^−^ vacancy defects (V_MA_ and V_I_), resulting in metallic lead defects under prolonged heating treatment and forming deep energy level defects. The carbonyl in PVP can suppress these vacancy defects and bond with excess Pb^2+^ by Lewis acid‐base interaction (Figure 3A). The schematic diagram of grain boundary suppression by PEAAc and defect passivation by PVP is illustrated in Figure 3H. Thus, the synergistic induction of PEAAc and PVP effectively reduces defects density and obtained a surface work function that matches the Cu electrode, thus improving the charge‐carriers transport.

### 2.4 X‐Ray Detector Performance

**Figure 4:**
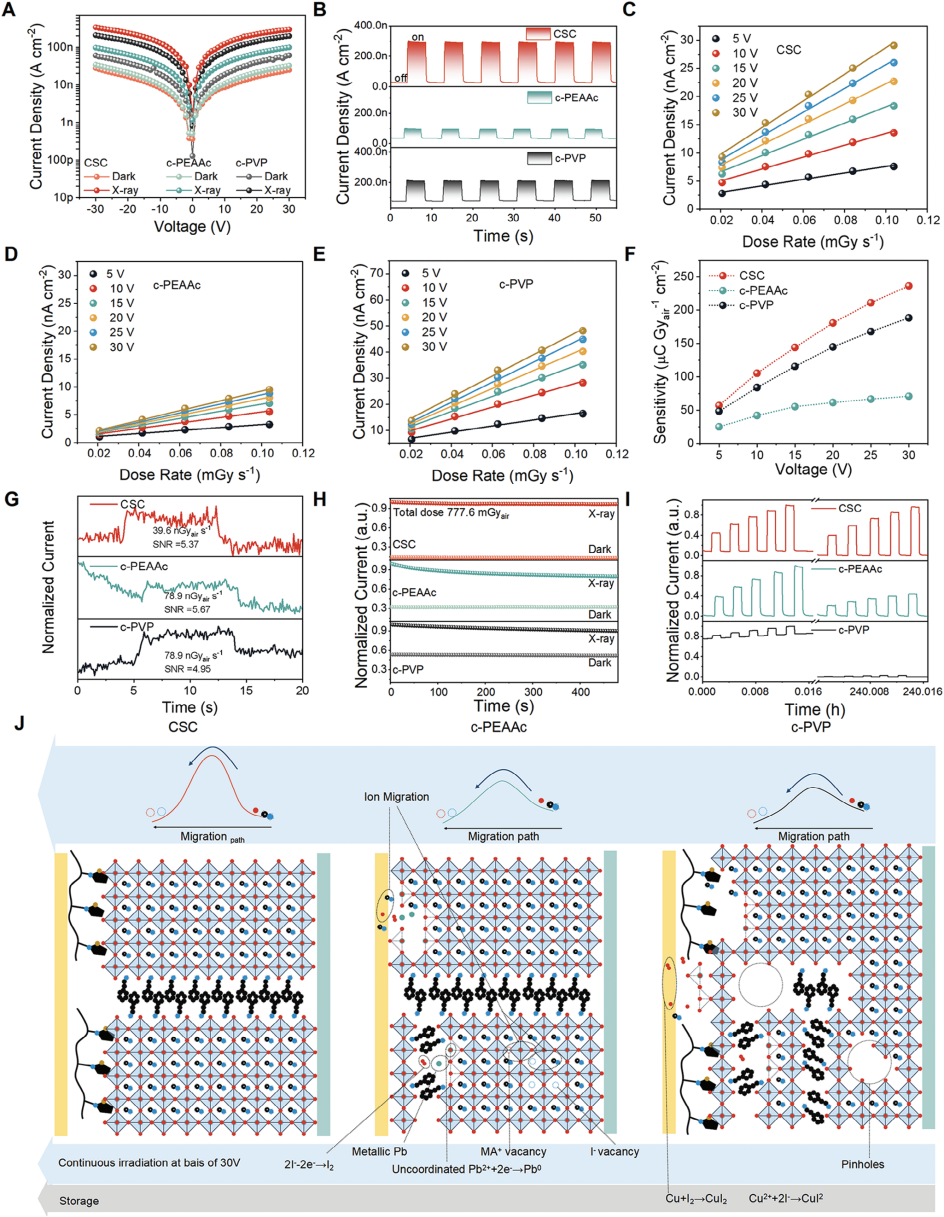
A) *J–V* curves of CSC, c‐PEAAc, and c‐PVP‐based X‐ray detectors under dark and X‐ray irradiation, the dose rate of X‐ray is 1.62 mGy_air_ s^−1^. B) *J–t* curves of the X‐ray detectors (1.62 mGy_air_ s^−1^, 30 V). C–E) X‐ray response net currents of CSC, c‐PEAAc, and c‐PVP‐based X‐ray detectors at various dose rates. F) The sensitivities at different voltages of the X‐ray detectors. G) *J–t* curves of the detectors at a low dose rate. H) X‐ray photocurrent and dark current of the X‐ray detectors were collected under continuous X‐ray irradiation with a total dose of 777.6 mGy_air_ at 30 V. I) The long‐term storage stability of X‐ray detectors stored in a glove box. J) Possible stability mechanism of CSC, c‐PEAAc, and c‐PVP‐based detectors.

To evaluate X‐ray detection performance, the current density (J)‐voltage (V) curves of CSC‐based detectors were measured in dark and under X‐ray (50 kVp) exposure, respectively, and compared with that of c‐PEAAc and c‐PVP‐based detectors, shown in **Figure** **4**A. Compared to the control devices, an obvious reduction of dark current density is observed in the detector using CSC‐based PEA_2_MA_4_Pb_5_I_16_ RP perovskite, which is beneficial to low X‐ray detection limit, due to the elimination of the pinholes in CSC‐based PEA_2_MA_4_Pb_5_I_16_ RP perovskite. Upon exposure to X‐ray irradiation, the CSC‐based detector possesses higher photocurrent density than the c‐PEAAc and c‐PVP‐based devices, which is attributed to the improved orientation and reduced trap‐state density that enhances the carrier transport. Correspondingly, under the same X‐ray dose rate and at 30 V, the CSC‐based device shows the highest on‐and‐off switching response, shown in Figure 4B. To obtain the sensitivity, the on/off photocurrent response under different dose rates and at different applied electric fields were gathered, shown in Figure 4C–E. The net X‐ray photocurrent exhibited a linear correlation with the X‐ray dose rate across different electric fields. The calculated X‐ray sensitivities at different applied biases are presented in Figure 4F. Notably, the sensitivity of the CSC‐based detector spans from 57 µC Gy_air_^−1^ cm^−2^ (under the bias of 5 V) to 236 µC Gy_air_^−1^ cm^−2^ (under the bias of 30 V), representing a 3.32‐fold increase compared to the c‐PEAAc‐based detector and a 1.25‐fold increase compared to the c‐PVP‐based detector.

The noise in radiation detector is generally dominated by dark current rather than other forms of noise, influencing the imaging efficacy of detectors particularly in low‐dose X‐ray conditions.^[^ ^44^ ^]^ To assess this impact, we conducted a systematic evaluation by measuring the response current of detectors under progressively diminishing X‐ray dose rates. As the dose rate decreased, the X‐ray‐induced signal weakened, eventually converging with the background noise. Remarkably, the CSC‐based device exhibits a discernible response signal amidst dark current noise, facilitating the discernment of the signal even at exceptionally low X‐ray doses (39.6 nGy_air_ s^−1^), as illustrated in Figure S13 (Supporting Information). In contrast, the c‐PEAAc‐based device demonstrated substantial dark current drift within a narrow range of current density, while the c‐PVP‐based device manifested fluctuating response signals, complicating the differentiation between signal and noise. Consequently, at an X‐ray dose rate of 39.6 nGy_air_ s^−1^, the signals of the c‐PEAAc and c‐PVP‐based devices amalgamate into the noise profile.

We then investigated the limit of detection (LoD) of the corresponding devices, wherein a diminished LoD signifies the potential for reduced radiation doses during X‐ray inspections, thereby amplifying the diagnostic value of imaging. LoD estimates were established by calculating the Signal‐to‐Noise Ratio (SNR) of the detectors. Figure 4G illustrates that the CSC‐based detector achieves an SNR of 5.37 under X‐ray irradiation at a dose rate of 39.6 nGy_air_ s^−1^. In contrast, the c‐PEAAc and c‐PVP‐based detectors necessitate a higher dose rate (78.9 nGy_air_ s^−1^) of X‐ray irradiation to attain SNRs of 5.67 and 4.95, respectively. The dose rate‐dependent SNR of detectors is elucidated in Figure S14 (Supporting Information), with the LoD derived from the curve when the SNR equals 3, providing detailed insights available in the Supporting Information. As anticipated, the synergistic effect of PEAAc and PVP in the CSC‐based detector achieved an impressively low LoD of 22.7 nGy_air_ s^−1^. This value is substantially below the requirements for general medical diagnostics (550 nGy_air_ s^−1^) and even surpasses the lowest reported LoD among quasi‐2D perovskite film detectors (69 nGy_air_ s^−1^).^[^ ^43^ ^]^ In contrast, the LoD for c‐PEAAc and c‐PVP devices were 69.0 and 64.9 nGy_air_ s^−1^, respectively. In summary, the collaboration of PEAAc and PVP in the CSC‐based device enhances the net X‐ray photocurrent while reducing dark current. This is attributed to heightened crystallinity, improved orientation, and diminished trap‐state density. Consequently, these improvements lead to enhanced carrier collection efficiency, a stable signal‐to‐noise response, and improvements in sensitivity and LoD when compared to control devices.

Stability is another important characteristic of X‐ray detectors. Both continuous irradiation stability and long‐term storage stability were evaluated, as shown in Figure 4H,I. Following 500 s of continuous X‐ray irradiation with a cumulative dose rate of 777.6 mGy_air_ in ambient conditions (23 °C, 69% RH), the CSC‐based detector demonstrates a sustained and stable dark current baseline alongside consistent X‐ray photocurrent output. In contrast, the c‐PEAAc‐based detector exhibits a notably attenuated X‐ray photocurrent, while the c‐PVP‐based detector demonstrates both deteriorated dark current and X‐ray photocurrent. Furthermore, after a long‐term storage period of 240 h in a glovebox environment (O_2_ < 0.1 ppm, H_2_O < 0.1 ppm), the CSC‐based detector experiences only marginal reductions in both X‐ray photocurrent and dark current, resulting in a negligible change in X‐ray sensitivity. Conversely, the c‐PEAAc detector shows a significant reduction in X‐ray photocurrent, resulting in a substantial loss of sensitivity. Meanwhile, the c‐PVP detector manifests a substantial decrease in both dark current and X‐ray photocurrent, leading to a pronounced sensitivity loss. This comparative analysis underscores the superior stability and sustained performance of the CSC‐based detector under prolonged exposure and storage conditions.

To probe the stability mechanism of CSC‐based detectors under continuous irradiation, we investigated the ion migration activation energy. This was achieved through the measurement of the electrical conductivity of films derived from CSC, c‐PEAAc, and c‐PVP at diverse temperatures, as depicted in Figure S15 (Supporting Information). The ion activation energy (E_a_) was subsequently determined by analyzing the slope of the ln(σT) −1 kT^−1^ relation, derived from the Nernst−Einstein function:^[^ ^45^ ^]^

(2) $$\sigma \;\left(T\right)=\frac{\sigma_{0}}{T}\;\exp\;(-\frac{E_{a}}{k_{B}T})$$

The ion migration activation energy (E_a_) of the CSC‐based detector was determined to be 669 meV, approximately twice that of c‐PEAAc (388 meV) and c‐PVP (334 meV). This discrepancy may be attributed to the presence of numerous MA^+^ vacancies in c‐PEAAc, resulting from the overreaction between PEAAc and MAI during prolonged heating. The subsequent generation of I^−^ vacancies occurs due to the excess I^−^ reacting to produce I_2_, leading to a significant number of uncoordinated Pb^2+^ ions that are oxidized into metallic Pb (Figure 3A). This phenomenon promotes ion migration of A‐ and X‐sites under X‐ray radiation and electric fields, resulting in unfavorable band bending during device operation and attenuating the X‐ray photocurrent. Moreover, the combined effect of ion migration and metallic Pb results in phase separation in c‐PEAAc, further reducing the current.^[^ ^46^ ^]^ In contrast, the X‐ray photocurrent attenuation in c‐PVP is less pronounced than in c‐PEAAc due to defects in c‐PVP are passivated by PVP. However, numerous grain boundaries and pinholes are present in c‐PVP, serving as important pathways for ion migration. For the CSC‐based detector, vacancy defects, metallic Pb, and grain boundaries are synergistically improved by PEAAc and PVP. Consequently, the CSC‐based sample shows the least attenuation of X‐ray photocurrent, indicating the improved stability under continuous irradiation.

During storage, the degradation of device performance is primarily attributed to the reaction of halogens with electrodes. In the c‐PEAAc‐based detector, residual I_2_ undergoes a redox reaction with the Cu electrode to form CuI_2_, leading to a series of chain effects that inhibit the generation and transport of X‐ray‐induced carriers, thereby resulting in a significant reduction in X‐ray photocurrent. In the c‐PVP‐based detector, PVP serves to block the contact of perovskites with electrodes. However, the presence of numerous grain boundaries in c‐PVP leads to the formation of a large number of weak dangling Pb‐I bonds,^[^ ^47^ ^]^ causing the fastest bulk decomposition, which in turn reduces the conductivity and the X‐ray photocurrent. Conversely, the CSC‐based detector exhibits fewer defects and grain boundaries, while simultaneously possessing PVP barrier layers, thereby demonstrating superior long‐term storage stability. The stability mechanism of the detectors is illustrated in Figure 4J.

**Figure 5:**
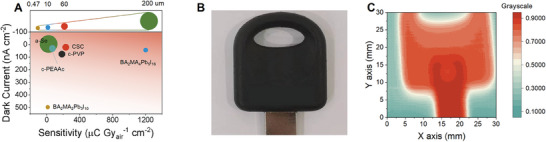
A) Comparison of the sensitivity and dark current density of commercial direct X‐ray detector of a‐Se, CSC, c‐PEAAc, c‐PVP, and reported quasi‐2D perovskites of BA_2_MA_2_Pb_3_I_10_ and BA_2_MA_4_Pb_5_I_16_ film‐based device.^[^ ^20^, ^22^, ^48^ ^]^ with different thicknesses. B,C) The photograph and corresponding X‐ray image of a metallic key partially wrapped in rubber.

To compare the performance of the X‐ray detector in this work with previous solution‐coating Q‐2D OIHPs and the state‐of‐the‐art commercial a‐Se X‐ray detectors, the key parameters, that is, sensitivity, LoD, thickness, and dark current, listed in Table S2 (Supporting Information). For a more intuitive comparison, sensitivity and dark current density are used to evaluate the overall performance of the detectors. In addition, the thickness of the film is positively correlated with absorbed X‐ray energy, thus the performance of X‐ray detectors with different thickness is also considered. The comprehensive result is presented in **Figure** **5**A, CSC‐based detector possesses high X‐ray sensitivity with low dark current, leading to ultralow LoD. In addition, due to the higher thickness, the X‐ray energy used for detection is the highest (Table S2, Supporting Information)

To demonstrate the capability of the imaging system, we scanned a metallic key partially hidden in plastics by a CSC‐based device (Figure 5B,C). For the metal and plastic, distinct color contrast is displayed on the images, suggesting that the detector can identify different materials of an object. Besides, we can clearly distinguish the hidden metal through the plastic and the different thickness of plastic parts by grayscale calculated by the induced photocurrent after X‐ray passing through the object. Interestingly, through X‐ray imaging, we find that the hidden metal appears like a tooth rather than simply a square. To evaluate the imaging quality, we conducted measurements of the spatial resolution (Figure S16, Supporting Information), defined as the spatial frequency value at the modulation transfer function (MTF) = 0.2.^[^ ^45^ ^]^ The determined spatial resolutions are 2.5, 1.0, and 0.8 lp mm^−1^ for the CSC, c‐PEAAc, and c‐PVP‐based detectors, respectively. The superior resolution observed in the CSC‐based X‐ray detector is attributed to its low LoD and high sensitivity, characteristics stemming from the presence of pure‐phase and vertically oriented quasi‐2D perovskites.

## 3 Conclusion

In conclusion, a novel carbonyl array‐synergized crystallization strategy has been successfully implemented to enhance the crystallinity, well‐width distribution, orientation, and trap state density of PEA_2_MA_4_Pb_5_I_16_ RP perovskite thick films obtained in situ from a solution. The improvements in these properties can be attributed to the large and dense intermediates as well as the enhanced binding energy of Pb^2+^ in the vertical direction, since carbonyls in PEAAc and PVP form Lewis acid‐base interaction with Pb^2+^ and the hydrogen bond with ‐NH_3_^+^. Moreover, the passivation of grain boundaries and ion vacancy defects by carbonyls in PEAAc and PVP, respectively, has resulted in reduced defect density. The thickness of the films processed by CSC strategy can reach more than half of a hundred micrometers, which exceeds that of previously reported PEA_2_MA_4_Pb_5_I_16_ RP perovskite films prepared through solution methods. The X‐ray sensitivity of the CSC‐based device has been found to be 3.32 and 1.25 times greater than that of the c‐PEAAc and c‐PVP‐based devices, respectively, which is also over 100 times higher than that of commercial a‐Se. The Limit of Detection (LoD) has been lowered to 22.7 nGy_air_ s^−1^, which satisfies the requirements of general medical diagnostics (550 nGy_air_ s^−1^). Furthermore, the CSC‐based detector has shown improved stability during long‐term X‐ray irradiation and long‐term storage, leading to higher resolution of X‐ray imaging.

## Conflict of Interest

The authors declare no conflict of interest.

## Author Contributions

Y.L. and H.C. conceived and designed the experiments. H.C. performed most of the experiments. Z.Z., B.Z., and W.H. assisted with the fabrication and characterization of the devices. G.Q. and Z.X. did the TRPL measurement and analyzed the data. S.Y. assisted with the KPFM measurement. Q.X. assisted in simulation calculation. H.C., Y.L., S.Y., and X.Y. took part in the discussions and gave important suggestions. H.C., Y.L., and S.Y. co‐wrote the manuscript.

## Supporting information

Supporting Information

## Data Availability

The data that support the findings of this study are available from the corresponding author upon reasonable request.
