# Supplementary material for: Vertically Oriented Quasi‐2D Perovskite Grown In‐Situ by Carbonyl Array‐Synergized Crystallization for Direct X‐Ray Detectors
Source: Adv Sci (Weinh). 2024 May 13;11(28):2309185. doi: 10.1002/advs.202309185 (PMC11267269; doi:10.1002/advs.202309185)
Supplement: Supplementary file 1 — Supporting Information [file ADVS-11-2309185-s001.pdf]

ADVANCED SCIENCE
Open Access

Supporting Information

for *Adv. Sci.*, DOI 10.1002/advs.202309185

Vertically Oriented Quasi-2D Perovskite Grown In-Situ by Carbonyl Array-Synergized Crystallization for Direct X-Ray Detectors

Huiwen Chen, Ziyao Zhu, Bo Zhao, Weixiong Huang, Geping Qu, Zong-Xiang Xu, Xue-Feng Yu, Quanlan Xiao, Shihe Yang* and Yunlong Li**

Supporting Information

Vertically oriented Quasi-2D Perovskite grown in-situ by carbonyl array-synergized crystallization for direct X-ray detectors

Huiwen Chen, Ziyao Zhu, Bo Zhao, Weixiong Huang, Geping Qu, Zong-Xiang Xu, Xue-Feng Yu, Quanlan Xiao,* Shihe Yang,* Yunlong Li*

Experimental Section

Materials: Methylammonium iodide (MAI, >99.5%) and 2-phenylethylammonium iodide (PEAI, >99.5%) were bought from Xi'an Polymer Light Co., Ltd. Lead iodide (PbI_2, >99.999%) was purchased from Advanced Election Technology Co., Ltd. 2-Phenethylamine (PEA, 98%) and Polyvinylpyrrolidone (PVP, MW. 58000) were supplied from Shanghai Macklin Biochemical Co., Ltd. Dimethyl sulfoxide (DMSO, AR), N, N-dimethylformamide (DMF, AR), ethanol and acetic acid (Ac, AR) were obtained from Shanghai Lingfeng Chemical Reagent Co., Ltd. All the reagents were used without further purification.

Synthesis of PEAAc: First, 4 mL PEA was injected in a solution mixed with 0.90 mL Ac and 6 mL ethanol and stirred in an ice water bath for 2 h. The resulting solution was placed in a rotary evaporator and evaporated at 80 °C for 1 h to remove ethanol. The product was washed three times with diethyl ether. Then the solid was dissolved with ethanol and was recrystallized with diethyl ether 3 times. Finally, the product was collected by rotary evaporator at 80 °C for 1 h.

Synthesis of 2D perovskite precursor: The precursor solution for CSC was prepared by

dissolving 50.7 mg of PEAAc, 133.5 mg of MAI, 322.7 mg of PbI_2, and 9.6 mg of PVP in 0.2 mL of a DMSO/DMF mixture solution (DMSO:DMF=1:4, v/v). This composition corresponds to the stoichiometry of $PEA_2MA_4Pb_5I_{16}$ according to the following equation:[1]

$$2PEAAc+5PbI_2+6MAI \rightarrow PEA_2MA_4Pb_5I_{16}+2MAAc\uparrow$$

The solution was stirred at 70 °C for 2 hours to ensure complete dissolution and formation of the precursor solution. Simultaneously, for the c-PEAAc based precursor, the same formula was utilized as for the CSC-based precursor, excluding the inclusion of PVP. As for the c-PVP-based precursor, 69.7 mg of PEAI, 89.0 mg of MAI, 322.7 mg of PbI_2, and 9.6 mg of PVP, corresponding to the composition of $PEA_2MA_4Pb_5I_{16}$ with 2 wt% PVP, were dissolved in the identical DMSO/DMF mixture solution as used for the CSC based precursor.

Device Fabrication: For X-ray detectors, the ITO substrate was washed with soap water, deionized water, ethanol, acetone, and isopropanol in turn. After 15 min ultraviolet-O3 treatment, the substrate was transferred to a nitrogen glove box. 100 μL of 2D perovskite precursor was loaded on the substrate with the size of 1.5 ×1.5 cm2 and bladed, with a speed of 7 mm s-1 and a gap between substrate and blade of 200 μm. Finally, the device was deposited with 50 nm Cu by the thermal evaporation system with a mask keeping an active area of 0.135 cm2. For the hole-only SCLC device, the substrate was firstly spin-coated by NiOx nanocrystal solution at 6000 r.p.m. for 30 s, which was then annealed at 120 °C for 10 min in air. The same precursor deposition procedure as X-ray detectors were performed. 50 nm Au was deposited by the thermal evaporation system. For the electron-only SCLC device, the same preparation process as X-ray detectors was performed, except that the final Cu electrode was replaced by

30 nm C_{60} and 80 nm Ag.

Materials Characterization: The morphology of the 2D perovskite thick films was obtained by scanning electron microscope (Gemini SEM 300, ZEISS). The surface roughness was measured by scanning force microscopy (Dimension Icon Bruker) under the atomic force microscopy subgroup in the air. The PeakForce KPFM suite of Dimension Icon allows surface potential measured in Kelvin probe force microscopy (KPFM) mode at the same area as the topographic obtained by AFM. The scanning area and scan rate were 5 μm × 5 μm and 0.498 Hz. The steady-state PL spectra were characterized by a PL spectrometer (Spectrofluorometer FS5, Edinburgh instruments). The crystal structure of thick films was acquired by an X-ray diffractometer (Smartlab3kw, RIGAKU) with a scanning speed of 10° min-1. The SCLC measurement was monitored by a semiconductor analyzer (FS-1/f noise, SINCEDA). The time-resolved photoluminescence (TRPL) spectra were obtained by a transient state fluorescence spectrometer (FLS1000, Edinburgh instruments) excitation at 365 nm. The GIWAXS data were obtained at 1W1A Diffuse X-ray Scattering Station, Beijing Synchrotron Radiation Facility (BSRF-1W1A). The X-ray beam photon energy is 10.9 keV and the incident angle is 0.5 °. The data is analyzed by GIWAXS-Tools. [2]

Device characterization: The X-ray detection performance was measured by a miniature X-ray tube (Mini-X2, Amptek) connected to the semiconductor analyzer. The acceleration voltage was 50 kV, and the current was varied from 40 to 200 μA. The dose rate of the X-ray was monitored by a dosimeter (X2 CT, Unfors Raysafe). The continuous irradiation stability was operated in the open air (23 °C, 69% RH). The samples for long-term storage stability measurement were firstly stored in a glove box

(O_2 < 0.1 ppm, H_2O < 0.1 ppm) and measured in the open air. The X-ray imaging measurement of CSC based device was performed by moving an object on an x-y stepping motor (GCD-402050M, Daheng Optics), with a scanning step of 0.75 mm. The semiconductor analyzer collected the current signal which was induced by the X-ray penetrating the object. A single pixel detector was prepared with an area of 1.5×1.5 mm^2. For modulation transfer function (MTF) calculation, single pixel imaging of the MTF standard test-pattern plate was carried out, with the motor scanning step of 0.02 mm and pixel size of 0.3 mm.

Computational details: Here, the density functional theory (DFT) implanted in the Vienna ab-initio simulation package (VASP) was adopted to model the binding energy of the Pb^{2+} ion adsorbed on the perovskite plane.[3,4] Based on the XRD results, the (111) and (202) planes were constructed to represent the experiment results. To reduce the amount of calculation, we only selected fragments of PVP molecule for calculation. The generalized gradient approximation (GGA) within the Perdew-Burke-Ernzerhof (PBE) formalism was performed to describe the exchange-correlation function[5]. The electron-ion interaction was described by the projector augmented wave (PAW) method[6]. A plane-wave cutoff energy of 500 eV was adopted for all the calculations. The convergence criterion for energy and force is set to 10^{-4} eV and 0.02 eV/Å, respectively. The Brillouin-zone integration is performed using Gamma only k-mesh for all the calculations, which can reduce the time consumption. The binding energy between the Pb ion and the substrate was calculated by the following equation:

$$E_{\text{Binding energy}}=E_{\text{all}}-E_{\text{Pb}}-E_{\text{substrate}}$$

where E_{all} is the total energy of the system, E_{Pb} and $E_{\text{substrate}}$ is the energy of the Pb atom and the substrate, respectively.

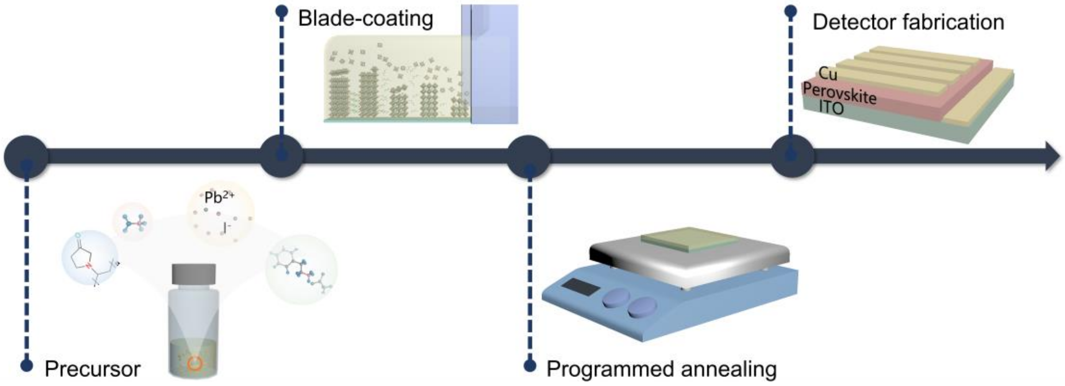

Figure S1. Schematic of X-ray detector fabricated by CSC precursor via blade coating process. The same method was used to fabricate c-PEAAc and c-PVP detectors.

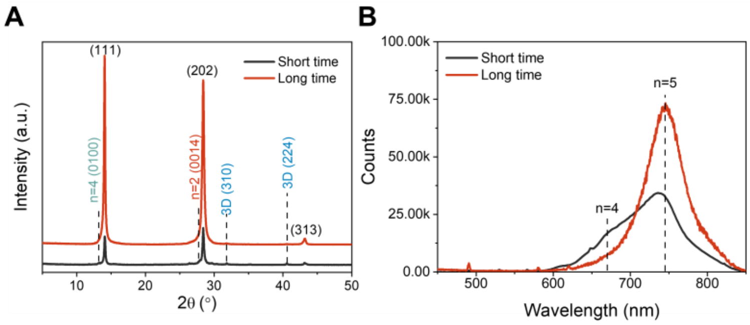

Figure S2. (A) XRD patterns and (B) PL spectra of films dried for a long time and a short time.

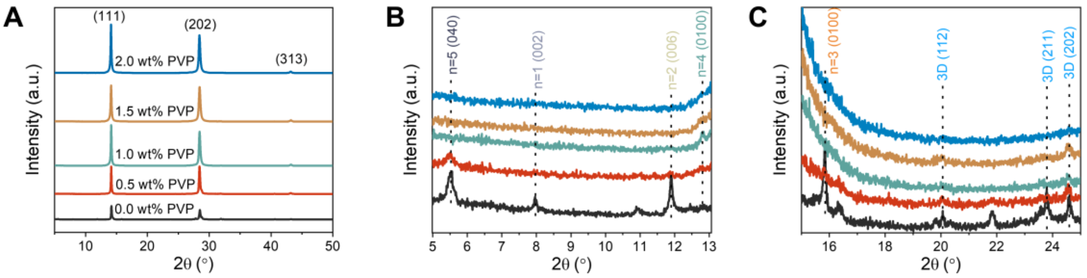

Figure S3. (A) XRD pattern of CSC based 2D perovskite films with different ratios of PVP. Enlarged image of 2θ at (B) 5-13 degrees and (C) 15-25 degrees.

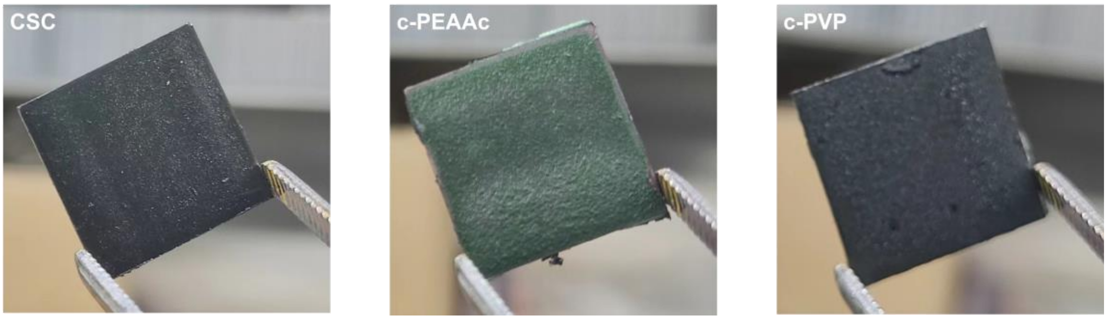

Figure S4. Photograph of CSC, c-PEAAc, and c-PVP based samples.

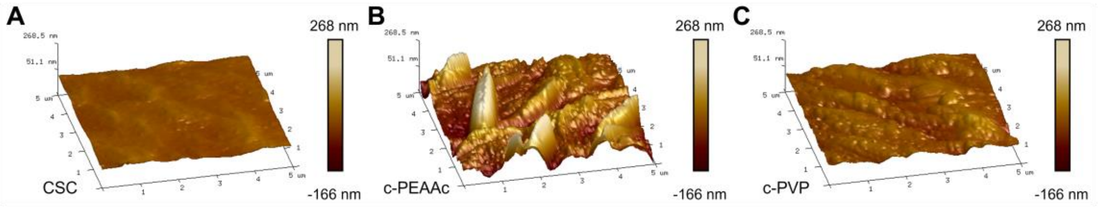

Figure S5. The topography maps (AFM) of (A) CSC, (B) c-PEAAc, and (C) c-PVP based layers.

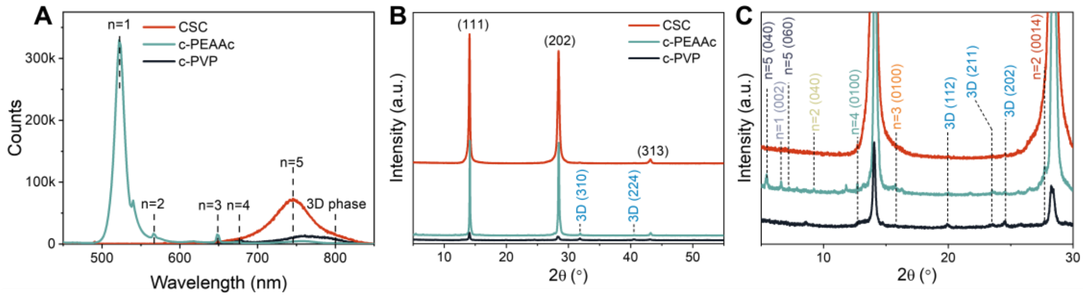

Figure S6 (A) PL spectra of CSC, c-PEAAc, and c-PVP based $PEA_2MA_4Pb_5I_{16}$ RP perovskite thick films, excited at 365 nm. (B, C) XRD patterns and corresponding

enlarged XRD patterns of CSC, c-PEAAc, and c-PVP based $PEA_2MA_4Pb_5I_{16}$ RP perovskite thick films.

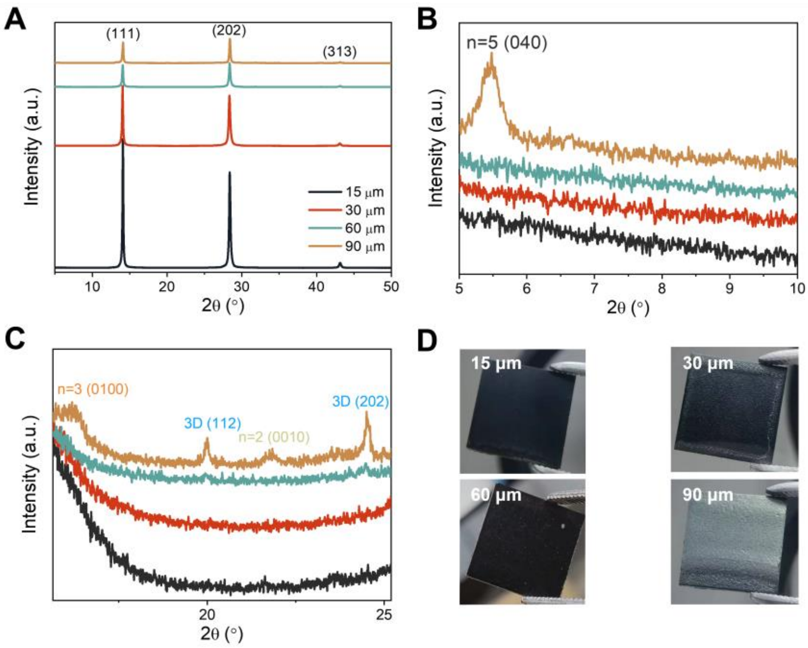

Figure S7. (A) XRD pattern of CSC based 2D perovskite films with different thicknesses. Enlarged image of 2θ at (B) 5-10 degrees and (C) 15-25 degrees. (D) Photograph CSC based 2D perovskite layers with different thicknesses.

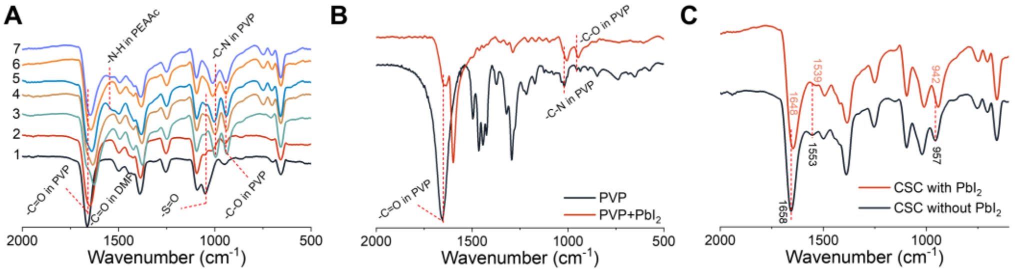

Figure S8. (A) FTIR spectra of 1-DMSO:DMF; 2-DMSO:DMF+PbI_2; 3-DMSO: DMF+PbI_2+PVP; 4-DMSO:DMF+PbI_2+PVP+PEAI; 5-DMSO:DMF+PbI_2+PVP+ MAI+PEAAc; 6-DMSO:DMF+PbI_2+PVP+PEAI+MAI; 7-DMSO:DMF+PbI_2+PVP +PEAAc+MAI. (B) PVP and PVP+PbI_2. (C) CSC precursor without and with PbI_2.

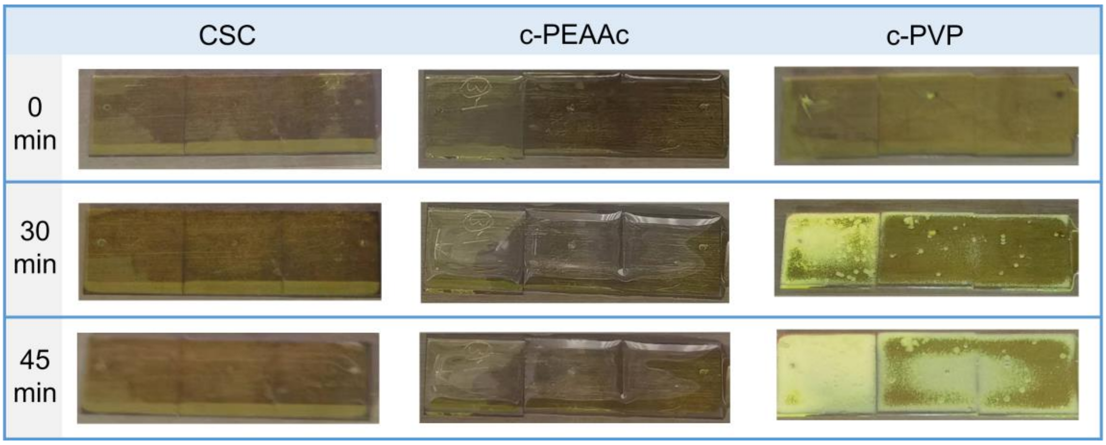

Figure S9. Photograph of CSC, c-PEAAc, and c-PVP based samples during the first 45 min of crystallization.

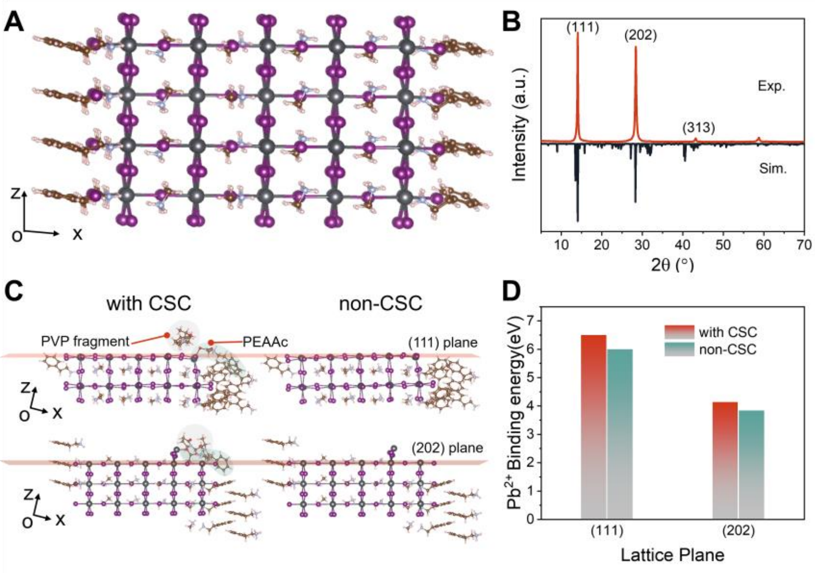

Figure S10. (A) Simulated structure of $PEA_2MA_4Pb_5I_{16}$. (B) Comparison of XRD pattern of simulated structure and experimental XRD result. (C) (111) and (202) plane structural models of CSC and non-CSC for theoretical calculation. (D) Binding energy of Pb^{2+} on (111) and (202) planes.

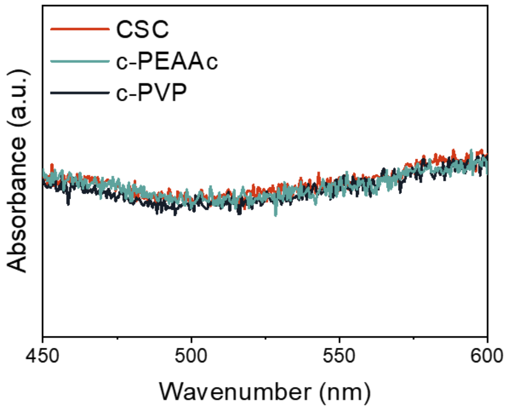

Figure S11. UV−Vis absorption spectra of CSC, c-PEAAc, and c-PVP based films.

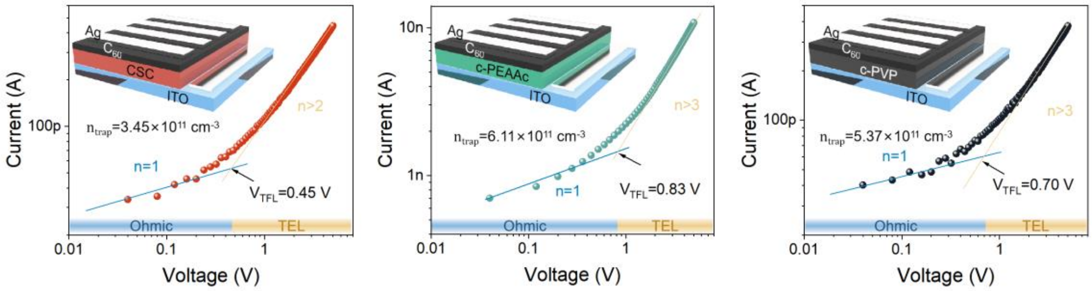

Figure S12. SCLC measurements of electron-only devices based on CSC, c-PEAAc and c-PVP.

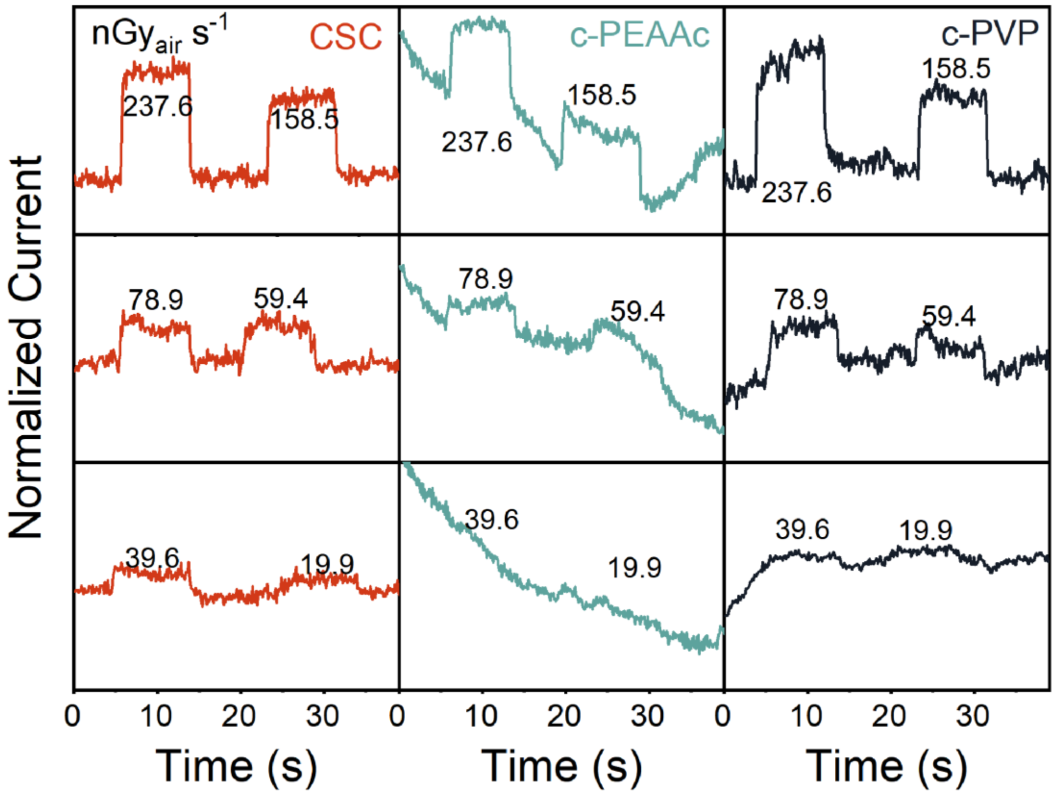

Figure S13. The response of CSC, c-PEAAc, and c-PVP based detectors to X-ray with various dose rates under a bias of 30 V.

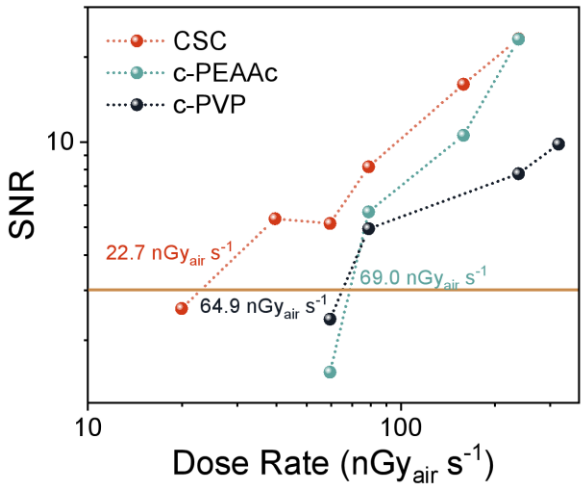

Figure S14. The dose rate-dependent SNR of CSC, c-PEAAc, and c-PVP based detectors.

The signal-to-noise ratio (SNR) was calculated by the Equation S1:

$$SNR=\frac{I_{signal}}{I_{noise}} \quad (S1)$$

I_{signal} is the net X-ray photocurrent, which is was derived by subtracting average X-ray photocurrent ($\bar{I}_{photo}$) by the average dark current ($\bar{I}_{dark}$). I_{noise} represents noise current, which is determined by standard deviation of photocurrent (Equation S2):[7]

$$I_{noise}=\sqrt{\frac{1}{N}\sum_{i}^{N}(I_i-\bar{I}_{photo})^2} \quad (S2)$$

To estimate the limit of detection (LoD), the SNR of the detectors as function of dose rate is plotted. The LoD derived from the curve when the SNR equals 3.

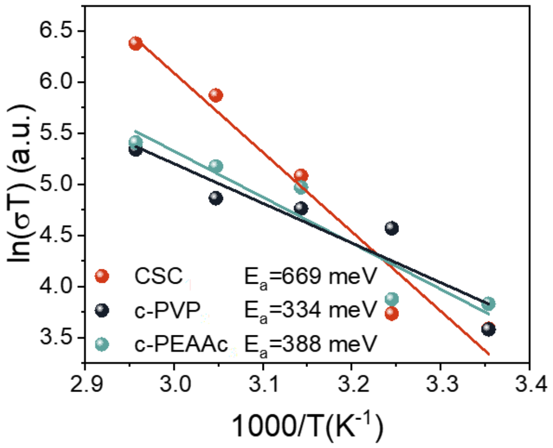

Figure S15. Results of temperature-dependent conductivity measurement of CSC, c-PVP, and c-PEAAc films.

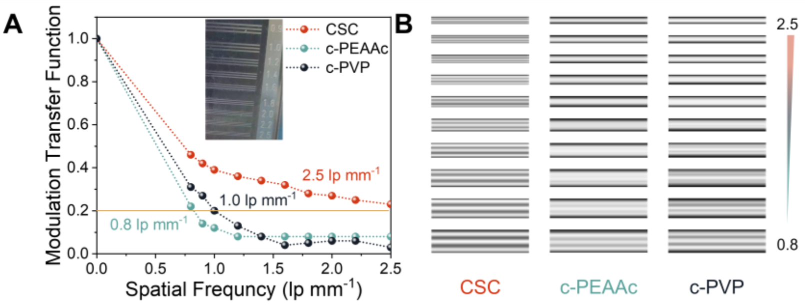

Figure S16. (A) MTF and (B) X-ray images of the MTF standard test-pattern plate of CSC, c-PEAAc, and c-PVP based detectors.

Table S1. The fitting results of TRPL of CSC, c-PEAAc, and c-PVP based thick films surface.

Sample	τ_1 (ns)	τ_2 (ns)	A_1	A_2	R^2
CSC	10.87	160.97	1226.45	436.13	0.9882
c-PEAAc	0.68	3.56	1203.79	11.40	0.9926
c-PVP	7.39	94.41	1454.07	337.10	0.9869

Table S2. X-ray detection performance of OIHP films prepared by solution method and amorphous selenium.

Device structure	Sensitivity ($\mu C\ Gy_{air}^{-1}\ cm^{-2}$)	LoD ($nGy_{air}\ s^{-1}$)	Electric field ($V\ mm^{-1}$)	Dark current ($nA\ cm^{-2}$)	X-ray energy	Thickness (μm)	Ref.
ITO/PTAA/$BA_2MA_2Pb_3I_{10}$/C_{60}/Au	13	-	0	~500	~20 kVp	0.47	[8]
ITO/PTAA/$BA_2MA_4Pb_5I_{16}$/C_{60}/BCP/Al_2O_3	1214	-	500	~50	30 kVp	10	[9]
a-Se	20	3800	10000	0.04	30 kVp	200	[10]
ITO/CSC based layer/Cu	236	22.7	500	25.8	50 kVp	60	This work
ITO/c-PEAAc based layer/Cu	71	69.0	500	34.4	50 kVp	60	This work
ITO/c-PVP based layer/Cu	188	64.9	500	77.3	50 kVp	60	This work

Reference

1. Liang C, Gu, H, Xia, Y, et al. Two-dimensional Ruddlesden–Popper layered perovskite solar cells based on phase-pure thin films. Nat. Energy. 2021; 6: 38-45.
2. Jianyao Huang, GIWAXS-Tools, Version [current version], https://gitee.com/swordshinehjy/giwaxs-script（accessed date）.
3. Kresse G,Furthmüller, J. Efficiency of ab-initio total energy calculations for metals and semiconductors using a plane-wave basis set. Comput. Mater. Sci. 1996; 6: 15-50.
4. Kresse G,Furthmüller, J. Efficient iterative schemes for ab initio total-energy calculations using a plane-wave basis set. Phys. Rev. B. 1996; 54: 11169.
5. Perdew JP, Burke, K,Ernzerhof, M. Generalized gradient approximation made simple. Phys. Rev. Lett. 1996; 77: 3865.

6. Blöchl PE. Projector augmented-wave method. Physical review B 1994; 50: 17953.
7. Liu Y, Xu, Z, Yang, Z, et al. Inch-size 0D-structured lead-free perovskite single crystals for highly sensitive stable X-ray imaging. Matter 2020; 3: 180-196.
8. Tsai H, Liu, F, Shrestha, S, et al. A sensitive and robust thin-film x-ray detector using 2D layered perovskite diodes. Sci. Adv. 2020; 6: eaay0815.
9. Tsai H, Shrestha, S, Pan, L, et al. Quasi-2D Perovskite Crystalline Layers for Printable Direct Conversion X-Ray Imaging. Adv. Mater. 2022; 34: 2106498.
10. Li Y, Adeagbo, E, Koughia, C, et al. Direct conversion X-ray detectors with 70 pA cm^{-2} dark currents coated from an alcohol-based perovskite ink. J. Mater. Chem. C 2022; 10: 1228-1235.
